# All-cause and cause-specific mortality in Scotland 1981–2011 by age, sex and deprivation: a population-based study

**DOI:** 10.1093/eurpub/ckz010

**Published:** 2019-02-13

**Authors:** Denise Brown, Mirjam Allik, Ruth Dundas, Alastair H Leyland

**Affiliations:** MRC/CSO Social and Public Health Sciences Unit, University of Glasgow, Glasgow, UK

## Abstract

**Background:**

Average life expectancy has stopped increasing for many countries. This has been attributed to causes such as influenza, austerity policies and deaths of despair (drugs, alcohol and suicide). Less is known on the inequality of life expectancy over time using reliable, whole population, data. This work examines all-cause and cause-specific mortality rates in Scotland to assess the patterning of relative and absolute inequalities across three decades.

**Methods:**

Using routinely collected Scottish mortality and population records we calculate directly age-standardized mortality rates by age group, sex and deprivation fifths for all-cause and cause-specific deaths around each census 1981–2011.

**Results:**

All-cause mortality rates in the most deprived areas in 2011 (472 per 100 000 population) remained higher than in the least deprived in 1981 (422 per 100 000 population). For those aged 0–64, deaths from circulatory causes more than halved between 1981 and 2011 and cancer mortality decreased by a third (with greater relative declines in the least deprived areas). Over the same period, alcohol- and drug-related causes and male suicide increased (with greater absolute and relative increases in more deprived areas). There was also a significant increase in deaths from dementia and Alzheimer’s disease for those aged 75+.

**Conclusions:**

Despite reductions in mortality, relative (but not absolute) inequalities widened between 1981 and 2011 for all-cause mortality and for several causes of death. Reducing relative inequalities in Scotland requires faster mortality declines in deprived areas while countering increases in mortality from causes such as drug- and alcohol-related harm and male suicide.

## Introduction

For several decades, many countries have seen steady increases in life expectancy. However recently, for some of these countries, there has been a flattening or decreasing trend in average life expectancy.^1^ This has been attributed to causes such as influenza epidemics among older people; austerity policies;^2^ and drug, alcohol and suicide deaths in deprived groups.^3^ In Scotland, between 1991 and 2001, mortality rates due to alcohol, drugs, suicide and assault increased for young men, particularly those in the most deprived areas.^4^ This has contributed to Scotland lagging behind other Western European countries when it comes to improvements in life expectancy.^5^

Inequalities in mortality have persisted in the UK for many years, with some suggesting that they are now at their highest level since 1921.^6^ Despite overall improvements in the health of the Scottish population, the gap between those with the best health and worst health persists and, for many health measures, the deprivation gap has widened. While some countries have struggled to reduce mortality inequalities, others have made considerable progress over recent years^7^ particularly in reducing absolute inequalities.^8^ Although relative reductions can be more difficult to achieve than absolute reductions when health is improving, it has been argued that ‘it is both possible and feasible to expect both absolute and relative measures of health inequalities to improve simultaneously given a conducive policy context’.^9^

Knowledge of how inequalities in mortality rates differ by key socio-economic factors, and are changing over the long-term, is crucial for monitoring public health and making progress on health inequalities.^10^ Previous work on inequalities in life expectancy and mortality has lacked reliable socio-economic data on the whole population across time.^1^ This paper uses population data from Scotland for the period 1981–2011 to give an overview of the changes to mortality rates over three decades, and describes current inequalities in all-cause and cause-specific mortality by age group, sex and area-level deprivation.

## Methods

### Population

We used census population estimates of the usually resident population on census day in Scotland. The estimated population was 5 178 248 in 1981 reducing to 5 106 135 in 1991 and to 5 062 011 in 2001 before rising to 5 295 403 in 2011.

### Mortality

**Figure 1:**
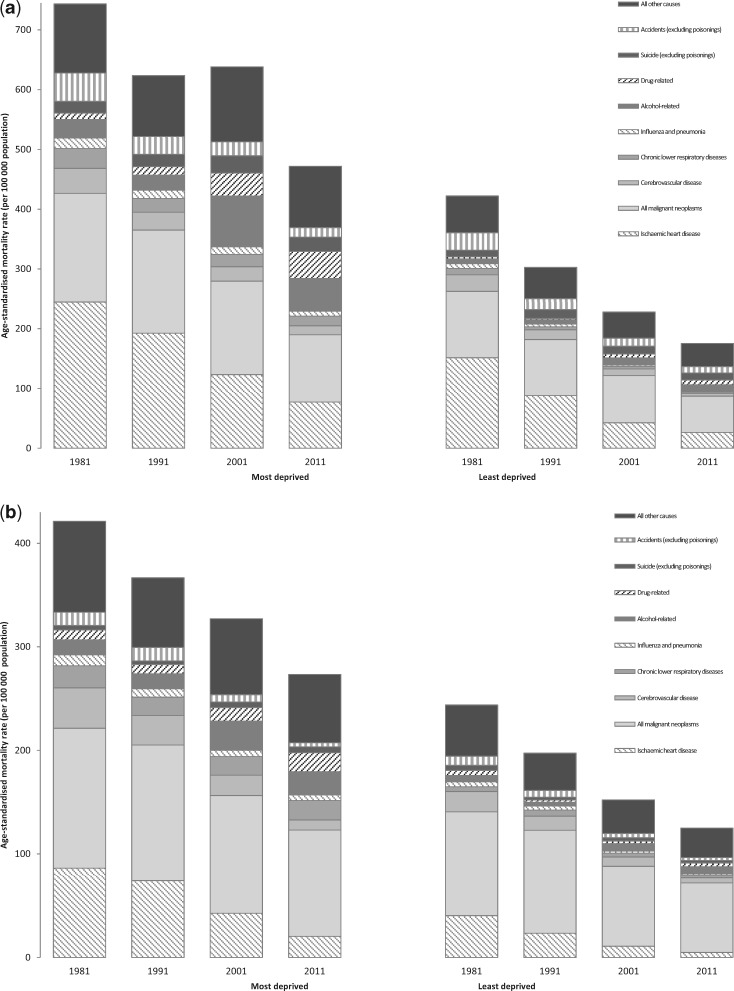
Stack plot showing all-cause mortality rates (per 100 000 population) for (a) Males, aged 0–64, in the most and least deprived fifths 1981–2011 and (b) females, aged 0–64, in the most and least deprived fifths 1981–2011

We obtained mortality information from vital events data held by National Records of Scotland for the periods 1980–82, 1991–92, 2000–02 and 2010–12. In total, there were 190 252 120 497, 171 592 and 161 807 deaths respectively. At least 99.6% of deaths within each period were linked to a Scottish postcode sector (i.e. ‘G12 8’). Note that there was a restructuring of some postcodes in the Scottish Grampian region in 1990. We therefore excluded deaths in 1990 from our analysis as it was not possible to assign death records to the new postcode sector boundaries. We looked at leading causes of death for males and females^11^ plus accidents and deaths of despair^3^ (drug- and alcohol-related harm and suicide). Together these causes of death accounted for 80% of the total deaths in Scotland in 1981 (and 74% of the total deaths in 2011). Causes of death (underlying) were coded in accordance with the International Classification of Diseases (ICD) using ICD-9 in 1980–82 and 1991–92 and ICD-10 in 2000–02 and 2010–12. Standard definitions of suicides^12^ and accidental deaths^13^ both record deaths due to drug and alcohol poisonings (already accounted for by drug- and alcohol-related deaths). To allow for the summing together of causes without overlap (as illustrated in figure 1), an alternative definition of suicides and accidental deaths that excludes poisonings is given in the Supplementary appendix.

### Area-level deprivation

Area-level deprivation was measured using Carstairs deprivation scores. Scores were created from four census variables (car ownership, male unemployment, overcrowding and low social class) in 1981, 1991, 2001 and 2011^14^^–^^17^ at the postcode sector level of geography. Postcode sectors were chosen as they were believed to be of sufficient size to reliably estimate rates of health events, including mortality.^14^ There were 1010 postcode sectors in Scotland in 2011 (average population size ∼5000 people, min: 52, max: 21 159).

### Analyses

Using the 2013 European standard population, data were aggregated into five-year age bands to calculate directly age-standardized all-cause and cause-specific mortality rates for the periods 1980–82, 1991–92, 2000–02 and 2010–12. Note that the upper age band of the 2013 European standard population is 95+, although using an upper age limit of 90+ is recommended.^18^ In this study population estimates up to age 90+ are available at postcode sector level in 1991, 2001 and 2011 but only to 85+ in 1981. For 1981, the split of males and females in age groups 85–89 and 90+ in postcode sectors is approximated using population estimates available at the wider Health Board (*n* = 15, pre-April 2006) level. Age-standardized mortality rates, for males and females separately, are presented for 15 year age groups (0–14, 15–29, 30–44, 45–59, 60–74 and 75+), ages 0–64 and for all ages. We show the change in rates between 1981 and 2011 (2011 rate minus the 1981 rate, divided by the 1981 rate and expressed as a percentage) but it is also straightforward to calculate the change in rates between decades and absolute rate changes over time. We divided the population at each census into population-weighted fifths (most to least deprived) according to Carstairs deprivation score of the postcode sector of residence. Mortality rates by deprivation fifths are presented for premature mortality, an important indicator of the overall health of the population. Since the starting point of our analysis is 1980–82, when average life expectancy was lower, we define premature mortality here as deaths of those aged 0–64.

## Results

### All-cause mortality

Male and female all-cause mortality rates decreased between 1981 and 2011 across all age groups (table 1). Female mortality was 26% lower than males (for all ages) in 2011, compared to 36% lower in 1981. Rates decreased most in the youngest age group (0–14 years) and at 45–74 years. For males, the smallest declines in all-cause mortality between 1981 and 2011 were in age groups 15–29 and 30–44 (reductions of 22 and 6%, respectively). The reductions were lower than in other age groups due in part to an increase in rates between 1991 and 2001 (from 98 to 113 deaths per 100 000 population in the 15–29 age group and from 174 to 204 deaths per 100 000 population in the 30–44 age group). The slowest rate of decline for females was in the 15–29 age group (12%). For all ages, male and female mortality decreased by 43 and 34%, respectively, between 1981 and 2011. Rates of decline were steeper in the 0–64 age group (48% for males and 43% for females) than for all ages.

**Table 1:** Age-standardized all-cause and cause-specific mortality rates (per 100 000 population) for males and females, 1980–82, 1991–92, 2000–02 and 2010–12 with % change showing overall percentage change in rates between 1981 and 2011

	Males					Females				
	Years				*% Change*	Years				*% Change*
Age	1980–82	1991–92	2000–02	2010–12	*81–11*	1980–82	1991–92	2000–02	2010–12	*81–11*
***All causes***										
0–14	121	77	53	38	−*68*	92	56	41	29	−*68*
15–29	98	98	113	77	−*22*	39	38	39	34	−*12*
30–44	209	174	204	196	−*6*	130	105	102	100	−*23*
45–59	1069	810	687	525	−*51*	617	492	416	347	−*44*
60–74	4310	3668	2890	2049	−*52*	2408	2147	1735	1377	−*43*
75+	15 710	13 993	11 678	9830	−*37*	10 881	9613	8783	7762	−*29*
***0–64***	***568***	***446***	***394***	***296***	−***48***	***327***	***266***	***224***	***186***	−***43***
***All ages***	***2424***	***2096***	***1739***	***1390***	−***43***	***1551***	***1357***	***1196***	***1028***	−***34***
***Ischaemic heart disease (ICD-9 410–414; ICD-10 I20–25)***										
0–14	0	0	0	0	*–*	0	0	0	0	*–*
15–29	1	1	1	1	*–*	0	0	0	0	*–*
30–44	46	28	20	16	−*65*	9	7	5	4	−*60*
45–59	432	287	160	101	−*77*	115	84	41	24	−*79*
60–74	1556	1285	736	374	−*76*	734	589	327	146	−*80*
75+	4054	3675	2667	1630	−*60*	2574	2388	1770	1025	−*60*
***0–64***	***200***	***139***	***79***	***46***	−***77***	***62***	***47***	***24***	***12***	−***81***
***All ages***	***720***	***607***	***398***	***232***	−***68***	***378***	***331***	***223***	***122***	−***68***
***Cancer (ICD-9 140–208; ICD-10 C00–97)***										
0–14	6	3	3	2	−*62*	4	4	2	2	*–*
15–29	9	6	6	5	−*45*	8	6	5	5	−*35*
30–44	37	32	25	21	−*42*	52	44	33	30	−*42*
45–59	282	253	208	158	−*44*	253	239	195	164	−*35*
60–74	1186	1186	1038	839	−*29*	672	739	672	617	−*8*
75+	2542	2742	2658	2533	*0*	1315	1412	1536	1545	*17*
***0–64***	***141***	***128***	***112***	***83***	−***41***	***119***	***113***	***93***	***80***	−***33***
***All ages***	***492***	***503***	***460***	***404***	−***18***	***294***	***309***	***297***	***282***	−***4***
***Stroke (ICD-9 430–438; ICD-10 I60–69, G45)***										
0–14	0	0	0	0	***–***	1	0	0	0	***–***
15–29	3	2	1	1	***–***	2	1	1	1	***–***
30–44	10	6	5	5	−***53***	11	7	5	3	−***76***
45–59	62	40	29	18	−***71***	55	38	25	14	−***74***
60–74	456	313	202	103	−***77***	339	231	150	77	−***77***
75+	2482	1992	1553	931	−***62***	2350	1858	1512	952	−***60***
***0–64***	***34***	***22***	***16***	***9***	−***73***	***29***	***19***	***13***	***7***	−***76***
***All ages***	***314***	***241***	***181***	***106***	−***66***	***282***	***215***	***167***	***102***	−***64***
***Chronic lower respiratory diseases (ICD-9 490–494, 496; ICD-10 J40–47)***										
0–14	1	0	0	0	*–*	1	0	0	0	***–***
15–29	1	1	1	0	*–*	1	1	1	0	***–***
30–44	3	1	1	2	*–*	2	2	1	1	***–***
45–59	37	21	17	14	−*62*	24	14	17	15	−***37***
60–74	256	194	171	122	−*52*	86	118	131	123	***42***
75+	918	885	739	561	−*39*	175	230	384	423	***142***
***0–64***	***21***	***13***	***11***	***8***	−***61***	***11***	***10***	***9***	***9***	−***21***
***All ages***	***133***	***116***	***99***	***74***	−***45***	***36***	***44***	***60***	***62***	***74***
***Influenza and pneumonia(ICD-9 480–488; ICD-10 J09–J18)***										
0–14	3	1	0	1	*–*	3	1	1	0	*–*
15–29	2	1	0	1	*–*	1	1	0	0	*–*
30–44	5	3	2	2	−*56*	3	2	1	2	*–*
45–59	20	15	12	8	−*62*	13	10	7	5	−*58*
60–74	161	104	56	43	−*73*	92	61	32	25	−*73*
75+	1766	1443	732	557	−*67*	1229	1036	580	436	−*65*
***0–64***	***12***	***9***	***6***	***4***	−***66***	***8***	***5***	***4***	***3***	−***65***
***All ages***	***191***	***151***	***78***	***59***	−***69***	***130***	***106***	***59***	***45***	−***65***
***Dementia and Alzheimer’s disease (ICD-9 290, 331.0; ICD-10 F01, F03, G30)***										
0–14	0	0	0	0	*–*	0	0	0	0	*–*
15–29	0	0	0	0	*–*	0	0	0	0	*–*
30–44	0	0	0	0	*–*	0	0	0	0	*–*
45–59	1	0	0	0	*–*	0	1	0	0	*–*
60–74	9	10	18	24	*173*	8	10	15	19	*130*
75+	105	209	423	683	*550*	126	241	528	804	*537*
***0–64***	***0***	***0***	***0***	***1***	***–***	***0***	***0***	***0***	***0***	***–***
***All ages***	***11***	***21***	***41***	***66***	***494***	***13***	***24***	***50***	***76***	***490***
***Alcohol-related (ICD-9 291, 303, 3050, 4255, 5710–5715, 5718–5719, E860; ICD-10 F10, K70, K73, X45, X65, Y15, G31.2, G62.1, I42.6, K29.2, K74.0–K74.2, K74.6, K86.0)***										
0–14	0	0	0	0	*–*	0	0	0	0	*–*
15–29	1	1	2	2	*–*	0	0	1	1	*–*
30–44	12	13	27	21	*85*	6	7	12	12	*91*
45–59	35	35	88	61	*76*	17	18	38	29	*69*
60–74	41	40	98	77	*87*	20	20	35	29	*41*
75+	22	22	38	37	*65*	12	11	14	13	*8*
***0–64***	***15***	***15***	***38***	***28***	***81***	***8***	***8***	***16***	***13***	***65***
***All ages***	***19***	***18***	***44***	***33***	***80***	***9***	***10***	***17***	***15***	***55***
***Drug-related (ICD-9 292, 304, 305.2–305.8, E850–858, E950.0–E950.5, E962.0, E980.0–E980.5; ICD-10 F11–16, F19, X40–44, X60–64, X85, Y10–14)***										
0–14	0	0	0	0	*–*	0	0	0	0	*–*
15–29	7	13	33	23	*233*	3	6	9	7	*101*
30–44	7	10	29	47	*537*	8	5	10	16	*94*
45–59	8	6	9	18	*128*	12	6	6	12	−*3*
60–74	5	4	3	6	*10*	8	4	5	5	−*37*
75+	5	4	3	4	−*4*	4	4	2	3	*–*
***0–64***	***6***	***7***	***17***	***22***	***287***	***7***	***5***	***6***	***9***	***38***
***All ages***	***6***	***7***	***14***	***19***	***227***	***6***	***4***	***6***	***8***	***23***
***Suicide (ICD-9 E950–959, 980–989; ICD-10 X60–84, Y87.0, Y10–34, Y87.2)***										
0–14	0	0	1	0	*–*	0	0	1	0	*–*
15–29	18	28	38	24	*36*	5	8	9	9	*71*
30–44	26	30	41	42	*64*	12	9	12	13	*9*
45–59	29	25	28	32	*8*	21	11	10	12	−*42*
60–74	26	21	23	15	−*44*	15	9	9	6	−*62*
75+	25	25	20	16	−*35*	12	10	7	5	−*60*
***0–64***	***20***	***22***	***28***	***25***	***28***	***11***	***8***	***9***	***9***	−***18***
***All ages***	***21***	***22***	***26***	***23***	***11***	***11***	***8***	***8***	***8***	−***28***
***Accidents (ICD-9 E800–929; ICD-10 V01-X59, Y85–86)***										
0–14	17	10	5	2	−***85***	9	7	3	1	−***93***
15–29	47	33	21	22	−***52***	10	9	6	6	−***34***
30–44	36	23	21	38	***6***	8	7	4	9	***6***
45–59	45	29	23	25	−***45***	17	9	8	11	−***35***
60–74	70	49	40	34	−***51***	40	26	17	18	−***55***
75+	338	256	189	202	−***40***	378	237	187	166	−***56***
***0–64***	***38***	***25***	***20***	***23***	−***39***	***12***	***9***	***6***	***7***	−***38***
***All ages***	***69***	***49***	***37***	***41***	−***41***	***49***	***32***	***24***	***23***	−***53***

Rates are shown for all ages and for broad age groups. Rates for 0–64 years and for all ages are shown in bold/italic. Mortality rates are rounded to the nearest whole number while % change shows the percentage change in actual (unrounded) rates. Note that % change is not calculated, for a particular age group, when cause-specific mortality rates are consistently <5 per 100 000 population over time.

### Cause-specific mortality

There were large declines in ischaemic heart disease deaths for males and females in all age groups (table 1). Cancer mortality rates decreased between 1981 and 2011 with most of the reduction in younger age groups. There was no reduction in rates of cancer mortality for males aged 75+ while rates for females aged 75+ increased by 17%. Rates for deaths due to stroke, influenza and pneumonia decreased by over 60% between 1981 and 2011. Mortality rates from chronic lower respiratory diseases almost halved for males between 1981 and 2011 while rates increased by 74% for females over the same period. Deaths from dementia and Alzheimer’s disease have increased substantially for males and females in the oldest age group. Rates of alcohol-related deaths and drug-related deaths also increased. For males, alcohol-related death rates increased by 80% and drug-related deaths rates by 227%. Most of the increase in drug-related deaths occurred in the 30–44 age group and rates for those aged 30+ have continued to increase over the last decade. For females, alcohol- and drug-related death rates increased by 55 and 23%, respectively, between 1981 and 2011. Mortality rates due to suicide increased for males aged 15–59 and for females aged 15–44; however, there was a noticeable decline (37%) in male suicide of those aged 15–29 between 2001 and 2011. Rates of accidental deaths reduced (by 41 and 53% for males and females, respectively) with reductions across most age groups. There was a modest increase in the rate of accidental deaths for males and females aged 30–44.

### Mortality by deprivation

There is a strong correlation between mortality and deprivation in Scotland, which has persisted over time (Supplementary appendix figure A1). Between 1981 and 2011, mortality rates for males aged 0–64 in the most deprived areas declined by 271 per 100 000 population compared to 247 per 100 000 population in the least deprived areas. In contrast, there was a relative reduction in rates of 37% in the most deprived areas compared to 58% in the least deprived (table 2). For females there were greater absolute declines in mortality rates in the most deprived areas (148 per 100 000 population compared to 119 per 100 000 population in the least deprived areas) but relative reductions were less (35% in the most deprived areas compared to 49% in the least deprived). For most causes of death, relative reductions in mortality rates between 1981 and 2011 were greater in the least deprived areas, compared to the most deprived (one exception is strokes in females where the relative reductions in rates over time were roughly comparable across deprivation groups). For alcohol- and drug-related deaths and male suicide, relative increases (or absolute increases where rates are relatively low) were higher in more deprived areas. Note that dementia and Alzheimer’s disease mortality rates are not shown in table 2 due to insufficient deaths at ages 0–64.

**Table 2:** Age-standardized all-cause and cause-specific mortality rates (per 100 000 population) for males and females aged 0–64 in 1980–82, 1991–92, 2000–02 and 2010–12 with % change showing overall percentage change in rates between 1981 and 2011

	Males					Females				
	Years				*% Change*	Years				*% Change*
	1980–82	1991–92	2000–02	2010–12	*81–11*	1980–82	1991–92	2000–02	2010–12	*81–11*
***All causes***										
Most deprived	743	624	638	472	−*37*	421	367	327	273	−*35*
2	617	502	448	346	−*44*	356	291	247	211	−*41*
3	550	424	370	278	−*49*	314	253	218	181	−*42*
4	507	383	314	234	−*54*	299	218	182	154	−*48*
Least deprived	422	303	228	175	−*58*	244	197	152	125	−*49*
***All Scotland***	***568***	***446***	***394***	***296***	−***48***	***327***	***266***	***224***	***186***	−***43***
***Ischaemic heart disease (ICD-9 410–414; ICD-10 I20–25)***										
Most deprived	245	192	123	77	−*68*	86	74	43	20	−*76*
2	220	160	94	55	−*75*	72	56	28	15	−*79*
3	199	136	77	44	−*78*	56	43	21	12	−*79*
4	186	117	63	35	−*81*	56	34	18	9	−*84*
Least deprived	152	88	43	26	−*83*	40	23	11	5	−*88*
***All Scotland***	***200***	***139***	***79***	***46***	−***77***	***62***	***47***	***24***	***12***	−***81***
***Cancer (ICD-9 140–208; ICD-10 C00–97)***										
Most deprived	182	173	157	113	−*38*	135	131	114	103	−*24*
2	151	140	122	95	−*37*	124	121	98	84	−*32*
3	137	121	107	80	−*42*	121	108	93	80	−*34*
4	121	114	100	74	−*38*	114	103	86	71	−*38*
Least deprived	111	94	79	61	−*45*	100	99	77	67	−*33*
***All Scotland***	***141***	***128***	***112***	***83***	−***41***	***119***	***113***	***93***	***80***	−***33***
***Stroke (ICD-9 430–438; ICD-10 I60–69, G45)***										
Most deprived	42	30	24	15	−*64*	39	28	20	10	−*75*
2	38	25	18	11	−*70*	33	20	16	9	−*73*
3	35	23	15	8	−*76*	27	21	13	7	−*75*
4	30	19	12	8	−*74*	28	14	10	5	−*82*
Least deprived	28	16	11	5	−*83*	20	14	9	5	−*74*
***All Scotland***	***34***	***22***	***16***	***9***	−***73***	***29***	***19***	***13***	***7***	−***76***
***Chronic lower respiratory diseases (ICD-9 490–494, 496; ICD-10 J40–47)***										
Most deprived	33	23	21	16	−*52*	21	18	18	19	−*12*
2	24	15	13	11	−*56*	12	10	12	12	*2*
3	20	13	11	8	−*62*	9	10	7	8	−*13*
4	15	9	6	5	−*65*	8	7	5	5	−*40*
Least deprived	11	5	4	3	−*77*	5	6	4	3	−*45*
***All Scotland***	***21***	***13***	***11***	***8***	−***61***	***11***	***10***	***9***	***9***	−***21***
***Influenza and pneumonia (ICD-9 480–488; ICD-10 J09-J18)***										
Most deprived	18	14	13	8	−*52*	11	8	6	5	−*50*
2	12	10	7	6	−*52*	10	5	4	4	−*62*
3	12	7	4	4	−*72*	7	4	3	2	−*68*
4	11	9	4	2	−*78*	8	4	2	2	−*77*
Least deprived	8	5	3	2	−*81*	5	4	2	1	−*69*
***All Scotland***	***12***	***9***	***6***	***4***	−***66***	***8***	***5***	***4***	***3***	−***65***
***Alcohol-related (ICD-9 291, 303, 3050, 4255, 5710–5715, 5718–5719, E860; ICD-10 F10, K70, K73, X45, X65, Y15, G31.2, G62.1, I42.6, K29.2, K74.0-K74.2, K74.6, K86.0)***										
Most deprived	31	25	85	55	*76*	14	14	28	23	*58*
2	14	19	48	36	*146*	9	11	20	16	*90*
3	12	15	31	25	*105*	6	7	15	12	*103*
4	11	11	24	17	*53*	5	6	11	9	*100*
Least deprived	8	7	11	10	*29*	6	3	7	6	*6*
***All Scotland***	***15***	***15***	***38***	***28***	***81***	***8***	***8***	***16***	***13***	***65***
***Drug-related (ICD-9 292, 304, 305.2–305.8, E850–858, E950.0-E950.5, E962.0, E980.0-E980.5; ICD-10 F11–16, F19, X40–44, X60–64, X85, Y10–14)***										
Most deprived	11	14	39	45	*327*	10	9	13	18	*90*
2	6	9	18	27	*381*	8	5	8	10	*26*
3	6	6	13	17	*198*	5	3	6	8	*64*
4	4	5	9	13	*266*	6	3	3	5	−*9*
Least deprived	3	2	6	8	*124*	5	2	2	4	−*25*
***All Scotland***	***6***	***7***	***17***	***22***	***287***	***7***	***5***	***6***	***9***	***38***
***Suicide (ICD-9 E950–959, 980–989; ICD-10 X60–84, Y87.0, Y10–34, Y87.2)***										
Most deprived	29	33	41	35	*19*	13	11	13	13	−*7*
2	20	23	33	31	*58*	12	8	10	10	−*17*
3	19	18	27	24	*25*	10	7	9	9	−*9*
4	17	20	22	22	*29*	9	5	6	8	−*17*
Least deprived	14	17	16	14	*0*	9	5	5	5	−*50*
***All Scotland***	***20***	***22***	***28***	***25***	***28***	***11***	***8***	***9***	***9***	−***18***
***Accidents (ICD-9 E800–929; ICD-10 V01-X59, Y85–86)***										
Most deprived	48	30	25	41	−*15*	13	14	7	14	*3*
2	40	28	22	23	−*42*	13	8	5	7	−*44*
3	38	25	20	21	−*45*	12	10	6	8	−*37*
4	35	25	19	17	−*53*	12	6	6	4	−*63*
Least deprived	30	18	13	15	−*49*	9	7	4	4	−*58*
***All Scotland***	***38***	***25***	***20***	***23***	−***39***	***12***	***9***	***6***	***7***	−***38***

Rates are shown by Carstairs deprivation score (most to least deprived fifth). Rates for the whole of Scotland are shown in bold/italic. Mortality rates are rounded to the nearest whole number while % change shows the percentage change in actual (unrounded) rates.

### Additional causes of death

Included in Supplementary appendix tables A1–A3 are breakdowns of cancer deaths by selected causes. Lung cancer mortality declined by 44% for males but increased for females in older age groups (Supplementary appendix table A1). Female breast cancer mortality rates decreased by 29% between 1981 and 2011 while prostate cancer mortality rates increased by 25% over the same period. There were reductions in rates of death due to colorectal cancer between 1991 and 2011 and stomach cancer between 1981 and 2011. Deaths due to lung cancer and colorectal cancer, in the under 65 s, decreased most in the least deprived areas (Supplementary appendix table A2) while for other cancers evidence of a deprivation gradient was less clear.

For suicides excluding poisonings (Supplementary appendix table A1), patterns were similar to all suicides although rates were lower across all time points and for all ages. For accidental deaths excluding poisonings, rates only differed to all accidental deaths in 2011 for age groups 15–74 (reflecting rule changes, with effect from 2011, to how ‘drug abuse’ deaths from ‘acute intoxication’ are coded. Previously recorded as ‘mental and behavioural disorders due to psychoactive substance use’, ‘drug abuse’ deaths from ‘acute intoxication’ are now coded as ‘poisoning’^19^). Compared to mortality rates for alcohol-related deaths, rates for alcohol-specific deaths^20^ tend to be slightly lower but with a similarly strong deprivation gradient.

### Contribution of specific causes to all-cause mortality rates

Figure 1 shows the contribution of the cause-specific mortality rates to all-cause mortality rates between 1981 and 2011 in the most and least deprived areas for (i) males and (ii) females, aged 0–64. To avoid causes of death overlapping, suicides and accidents excluding poisonings were included in the plots. Rates for these nine causes of death (taken from table 2 and Supplementary appendix table A2) sum to the overall all-cause mortality rate together with the rate for ‘all other causes’. It is clear that despite reductions in mortality rates between 1981 and 2011 rates in the most deprived areas in 2011 remained higher than in the least deprived areas in 1981. As the contribution from deaths due to cancer, circulatory and respiratory causes to all-cause mortality declines over time, we can see the increasing contribution from alcohol- and drug-related causes.

## Discussion

All-cause mortality rates in Scotland have declined over the last 30 years. The (absolute and relative) gap between male and female mortality rates has narrowed and there have been reductions in mortality rates across all age groups. We have shown that death rates fell for males aged 15–44 between 2001 and 2011, following a period of increase between 1991 and 2001.^4^ Of all age groups, the greatest reduction in male mortality between 2001 and 2011 occurred in the 15–29 year age group (32% reduction) while the smallest decline was in the 30–44 year age group (4% reduction). The causes of death driving this finding appear to be drug-related deaths and suicide both of which reduced in the 15–29 year age group between 2001 and 2011 but rose in the 30–44 year age group; suggesting a possible cohort effect for these causes of death.^21^^,^^22^ Despite reductions in all-cause mortality rates across deprivation fifths, rates were higher in the most deprived areas in 2011 than in the least deprived areas in 1981. There were greater absolute reductions in all-cause mortality in the most deprived areas between 1981 and 2011, but relative reductions were highest in the least deprived areas. Overall, absolute inequalities in all-cause mortality in Scotland narrowed between 1981 and 2011 while relative inequalities widened. Narrowing absolute inequalities and widening relative inequalities have been reported elsewhere.^7^^,^^9^^,^^23^ Within Europe, some Southern European countries have been successful in reducing relative inequalities.^24^ This has been attributed to smaller inequalities in smoking and alcohol-related deaths in these countries.

For many causes of death (ischaemic heart disease, cancer, stroke, chronic lower respiratory diseases, influenza and pneumonia and accidents), premature mortality rates decreased over time but generally at a higher rate in the least deprived areas compared to the most deprived. Conversely, premature mortality rates for drug- and alcohol-related deaths and male suicide increased over time with larger increases in more deprived areas. Faster declines in mortality in deprived areas for leading causes of death and at least a flattening of deaths due to drug- and alcohol-related mortality and male suicide would help to decrease widening relative inequalities and reduce premature mortality in Scotland. Recent work has shown that lifespan variation, summarizing the inequality in age of death, is generally lower in countries with higher life expectancy, and countries that have been able to reduce lifespan variation are those which have been more successful in reducing premature mortality.^25^ A focus on reducing premature deaths in Scotland could lead to lower lifespan variation, in line with other countries,^26^ and an increase in average life expectancy.

The increase in deaths at younger ages due to alcohol, drugs and suicide, observed in Scotland between 1991 and 2001,^4^ has since been seen in the USA.^3^ There appears to be little effect of an upturn in mortality rates due to deaths of despair so far in Europe, but some have suggested the possibility of a lag.^27^ In Scotland, suicide and alcohol-related mortality rates declined after 2001 but remain above 1991 levels. Rates of drug-related mortality have continued to rise between 2001 and 2011 with Scotland having the highest rate of drug-related deaths in Europe.^28^ At older ages, deaths due to heart disease, cancer and stroke continue to decline while mortality from dementia and Alzheimer’s disease has increased significantly over each of the last three decades, a trend that looks set to continue. In Europe, only Finland has higher death rates due to dementia and Alzheimer’s disease.

Following a period of stalling life expectancy rates in Scotland, latest estimates suggest that there has been a small decrease in life expectancy for both males and females.^29^ This has also been observed in the rest of the UK and the USA.^1^ While other high income countries have also seen recent small declines in average life expectancy, which tend to be driven by mortality (such as respiratory, cardiovascular and Alzheimer’s disease) at older ages, the declines have been offset by previous increases in life expectancy. In the USA almost all the decline was attributable to drug abuse and external causes.^1^ Scotland not only faces increasing drug-related mortality at younger ages but also increasing deaths at older ages from dementia and Alzheimer’s disease.

### Strengths and limitations

This is a large population study examining trends in mortality over a 30 year period allowing assessment of overall progress towards lower mortality rates and reducing inequalities. Over time, there have been changes to how some deaths are coded. An ICD revision in 1999 has meant that some causes of deaths have been assigned to different categories. As a result, any observed changes between 1991 and 2001 (or overall change between 1981 and 2011) may appear larger than it actually is. This should not however affect recent changes in mortality rates between 2001 and 2011. The Carstairs deprivation measure has been subject to some criticism about the variables included in its construction and their appropriateness for use today,^30^ however, recent work has shown the relative usefulness of Carstairs deprivation scores compared to other deprivation indices.^31^

### Conclusions and policy implications

Despite all-cause mortality rates in Scotland decreasing over the last three decades, rates in the most deprived areas in 2011 remained higher than in the least deprived areas some 30 years previously. For several causes of death, mortality rates reduced most in the least deprived areas. For causes of death due to drug- and alcohol-related harm and male suicide, rates increased at a faster pace in deprived areas. Overall we saw a reduction in absolute inequalities in all-cause mortality. Although this is important from a public health perspective, so too is progress in the reduction of relative inequalities,^32^ brought about by faster improvements in the health and mortality outcomes of those in the most disadvantaged groups.

Medical advances have been responsible for vast improvements in mortality rates for many causes of death over the last three decades; however, the increases in deaths due to drug and alcohol abuse and suicide need upstream preventative policies which tackle the root causes of these deaths. Deaths from these causes have offset improvements in young adult age groups. Encouragingly, rates have started to decrease over the last 10 years in some age groups where they had previously risen and recent Government policies^33^^–^^36^ may help to drive these rates down further.

## Supplementary Material

ckz010_Supplementary_Appendix
