# Supplementary material for: All-cause and cause-specific mortality in Scotland 1981–2011 by age, sex and deprivation: a population-based study
Source: Eur J Public Health. 2019 Feb 13;29(4):647–55. doi: 10.1093/eurpub/ckz010 (PMC6660111; doi:10.1093/eurpub/ckz010)
Supplement: ckz010_Supplementary_Appendix [file ckz010_supplementary_appendix.docx]

**Supplementary appendix**

| 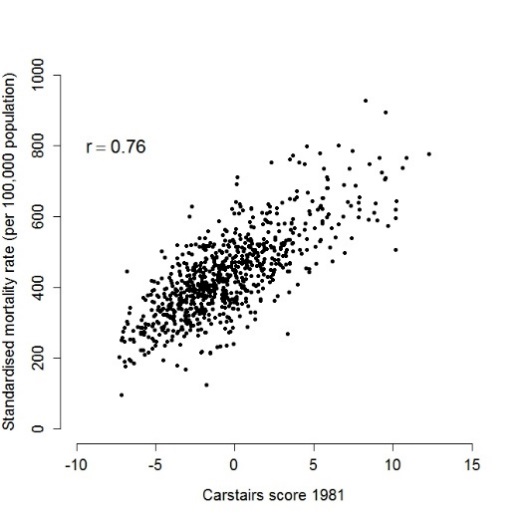 | 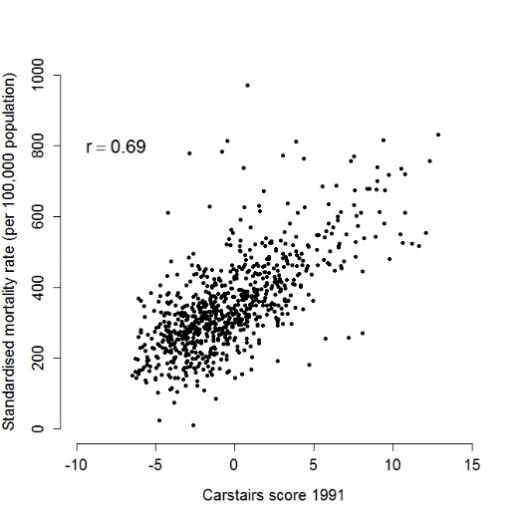 |
| --- | --- |
| (a) | (b) |
| 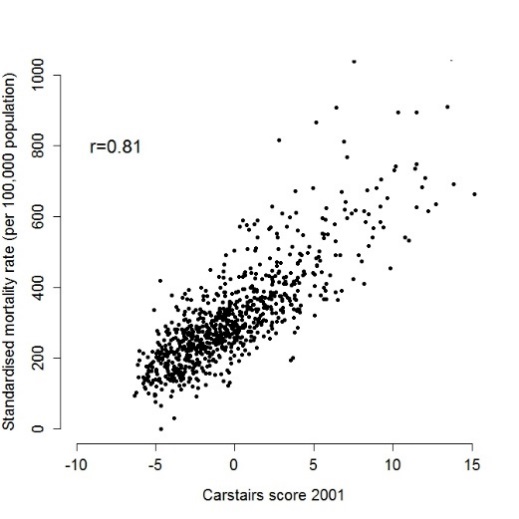 | 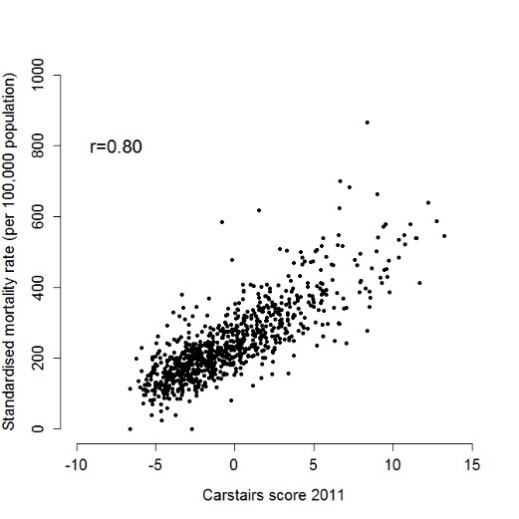 |
| (c) | (d) |

Figure A1: Age-standardised all-cause mortality rates (per 100,000 population) for all people aged 0-64 years by (a) 1981 Carstairs scores (deaths 1980-82) (b) 1991 Carstairs scores (deaths 1991-92) (c) 2001 Carstairs scores (deaths 2000-02) and (d) 2011 Carstairs scores (deaths 2010-12) in Scottish postcode sectors (with a population of at least 1,000 residents). Pearson’s correlation coefficient (r) is given.

Table A1: Age-standardised cause-specific mortality rates (per 100,000 population) for some additional causes of death for males and females, 1980-82, 1991-92, 2000-02, and 2010-12 with % change showing overall percentage change in rates between 1981 and 2011. Rates are shown for all ages and for broad age groups

|  | Males | | | | |  | Females | | | | |
| --- | --- | --- | --- | --- | --- | --- | --- | --- | --- | --- | --- |
|  | Years | | | | ***% Change*** |  | Years | | | | ***% Change*** |
| Age | 1980-82 | 1991-92 | 2000-02 | 2010-12 | ***81-11*** |  | 1980-82 | 1991-92 | 2000-02 | 2010-12 | ***81-11*** |
| ***Lung cancer (ICD-9 162; ICD-10 C33-34)*** | | | | | | | | | | | |
| 0-14 | 0 | 0 | 0 | 0 | *-* |  | 0 | 0 | 0 | 0 | *-* |
| 15-29 | 0 | 0 | 0 | 0 | *-* |  | 0 | 0 | 0 | 0 | *-* |
| 30-44 | 8 | 5 | 3 | 3 | *-60* |  | 4 | 4 | 3 | 2 | *-* |
| 45-59 | 122 | 84 | 59 | 40 | *-67* |  | 53 | 45 | 41 | 39 | *-26* |
| 60-74 | 530 | 482 | 350 | 259 | *-51* |  | 142 | 203 | 197 | 200 | *41* |
| 75+ | 825 | 801 | 677 | 601 | *-27* |  | 131 | 227 | 305 | 371 | *184* |
| ***0-64*** | ***60*** | ***45*** | ***32*** | ***22*** | ***-64*** |  | ***23*** | ***22*** | ***20*** | ***20*** | ***-16*** |
| ***All ages*** | ***189*** | ***170*** | ***131*** | ***106*** | ***-44*** |  | ***47*** | ***64*** | ***69*** | ***75*** | ***60*** |
| ***Breast cancer (ICD-9 175; ICD-10 C50)*** | | | | | | | | | | | |
| 0-14 |  |  |  |  |  |  | 0 | 0 | 0 | 0 | *-* |
| 15-29 |  |  |  |  |  |  | 0 | 1 | 0 | 0 | *-* |
| 30-44 |  |  |  |  |  |  | 19 | 15 | 11 | 9 | *-53* |
| 45-59 |  |  |  |  |  |  | 74 | 68 | 51 | 39 | *-48* |
| 60-74 |  |  |  |  |  |  | 110 | 111 | 90 | 75 | *-32* |
| 75+ |  |  |  |  |  |  | 179 | 211 | 193 | 177 | *-1* |
| ***0-64*** |  |  |  |  |  |  | ***32*** | ***29*** | ***22*** | ***17*** | ***-46*** |
| ***All ages*** |  |  |  |  |  |  | ***54*** | ***55*** | ***45*** | ***38*** | ***-29*** |
| ***Prostate cancer (ICD-9 185; ICD-10 C61)*** | | | | | | | | | | | |
| 0-14 | 0 | 0 | 0 | 0 | *-* |  |  |  |  |  |  |
| 15-29 | 0 | 0 | 0 | 0 | *-* |  |  |  |  |  |  |
| 30-44 | 0 | 0 | 0 | 0 | *-* |  |  |  |  |  |  |
| 45-59 | 5 | 4 | 6 | 4 | *-19* |  |  |  |  |  |  |
| 60-74 | 68 | 81 | 80 | 64 | *-7* |  |  |  |  |  |  |
| 75+ | 320 | 418 | 455 | 443 | *38* |  |  |  |  |  |  |
| ***0-64*** | ***3*** | ***3*** | ***4*** | ***3*** | ***-*** |  |  |  |  |  |  |
| ***All ages*** | ***41*** | ***52*** | ***55*** | ***51*** | ***25*** |  |  |  |  |  |  |
| ***Colorectal cancer (ICD-9 153, 154.0-154.1; ICD-10 C18-20)*** | | | | | | | | | | | |
| 0-14 | 0 | 0 | 0 | 0 | *-* |  | 0 | 0 | 0 | 0 | *-* |
| 15-29 | 0 | 0 | 0 | 1 | *-* |  | 0 | 0 | 0 | 0 | *-* |
| 30-44 | 3 | 4 | 2 | 2 | *-* |  | 3 | 2 | 2 | 2 | *-* |
| 45-59 | 28 | 30 | 22 | 14 | *-48* |  | 21 | 22 | 14 | 13 | *-38* |
| 60-74 | 116 | 119 | 112 | 82 | *-29* |  | 84 | 81 | 57 | 50 | *-41* |
| 75+ | 345 | 353 | 307 | 282 | *-18* |  | 263 | 209 | 193 | 172 | *-35* |
| ***0-64*** | ***13*** | ***14*** | ***12*** | ***8*** | ***-40*** |  | ***11*** | ***10*** | ***6*** | ***7*** | ***-38*** |
| ***All ages*** | ***57*** | ***58*** | ***51*** | ***42*** | ***-25*** |  | ***43*** | ***37*** | ***30*** | ***27*** | ***-37*** |
| ***Stomach cancer (ICD-9 151; ICD-10 C16)*** | | | | | | | | | | | |
| 0-14 | 0 | 0 | 0 | 0 | *-* |  | 0 | 0 | 0 | 0 | *-* |
| -15-29 | 0 | 0 | 0 | 0 | *-* |  | 0 | 0 | 0 | 0 | *-* |
| 30-44 | 3 | 2 | 1 | 1 | *-* |  | 1 | 1 | 2 | 1 | *-* |
| 45-59 | 21 | 14 | 9 | 6 | *-72* |  | 9 | 6 | 4 | 3 | *-71* |
| 60-74 | 84 | 77 | 50 | 30 | *-64* |  | 44 | 32 | 21 | 11 | *-74* |
| 75+ | 263 | 174 | 135 | 104 | *-60* |  | 137 | 92 | 71 | 46 | *-67* |
| ***0-64*** | ***11*** | ***7*** | ***5*** | ***3*** | ***-76*** |  | ***4*** | ***3*** | ***2*** | ***1*** | ***-69*** |
| ***All ages*** | ***43*** | ***31*** | ***23*** | ***16*** | ***-63*** |  | ***22*** | ***15*** | ***11*** | ***7*** | ***-69*** |
| ***Other cancers (all cancers excluding those above)*** | | | | | | | | | | | |
| 0-14 | 6 | 3 | 3 | 2 | *-61* |  | 4 | 4 | 2 | 2 | *-* |
| 15-29 | 8 | 6 | 6 | 4 | *-48* |  | 6 | 5 | 4 | 4 | *-42* |
| 30-44 | 23 | 21 | 18 | 16 | *-33* |  | 24 | 22 | 15 | 16 | *-31* |
| 45-59 | 101 | 121 | 113 | 94 | *-7* |  | 97 | 97 | 85 | 70 | *-27* |
| 60-74 | 370 | 428 | 447 | 404 | *9* |  | 292 | 311 | 307 | 280 | *-4* |
| 75+ | 851 | 996 | 1083 | 1103 | *30* |  | 606 | 672 | 775 | 779 | *28* |
| ***0-64*** | ***53*** | ***58*** | ***59*** | ***48*** | ***-10*** |  | ***49*** | ***48*** | ***43*** | ***36*** | ***-28*** |
| ***All ages*** | ***166*** | ***191*** | ***200*** | ***189*** | ***14*** |  | ***129*** | ***138*** | ***142*** | ***135*** | ***5*** |
| ***Suicide (excluding alcohol and drug poisonings, ICD-9 E950.6-959, E980.6-989; ICD-10 X64-84, Y87.0, Y16-34, Y87.2)*** | | | | | | | | | | | |
| 0-14 | 0 | 0 | 1 | 0 | *-* |  | 0 | 0 | 1 | 0 | *-* |
| 15-29 | 12 | 18 | 28 | 17 | *41* |  | 2 | 3 | 5 | 5 | *178* |
| 30-44 | 19 | 21 | 31 | 28 | *46* |  | 4 | 4 | 5 | 6 | *39* |
| 45-59 | 22 | 20 | 22 | 24 | *9* |  | 10 | 5 | 5 | 5 | *-48* |
| 60-74 | 21 | 17 | 19 | 11 | *-50* |  | 8 | 5 | 4 | 3 | *-65* |
| 75+ | 20 | 20 | 17 | 12 | *-40* |  | 8 | 7 | 5 | 3 | *-66* |
| ***0-64*** | ***15*** | ***16*** | ***21*** | ***18*** | ***22*** |  | ***5*** | ***3*** | ***4*** | ***4*** | ***-10*** |
| ***All ages*** | ***16*** | ***16*** | ***21*** | ***16*** | ***4*** |  | ***5*** | ***4*** | ***4*** | ***4*** | ***-26*** |
| ***Accidents (excluding alcohol and drug poisonings, ICD-9 E800-849, E861-929; ICD-10 V01-X39, X46-59, Y85, Y86)*** | | | | | | | | | | | |
| 0-14 | 17 | 10 | 5 | 2 | *-85* |  | 9 | 7 | 3 | 1 | *-93* |
| 15-29 | 46 | 32 | 20 | 12 | *-75* |  | 9 | 9 | 6 | 4 | *-59* |
| 30-44 | 35 | 23 | 20 | 14 | *-59* |  | 7 | 6 | 4 | 2 | *-67* |
| 45-59 | 44 | 28 | 23 | 15 | *-65* |  | 15 | 9 | 7 | 5 | *-66* |
| 60-74 | 69 | 49 | 40 | 31 | *-56* |  | 39 | 26 | 17 | 15 | *-61* |
| 75+ | 337 | 256 | 189 | 202 | *-40* |  | 378 | 236 | 187 | 165 | *-56* |
| ***0-64*** | ***37*** | ***25*** | ***19*** | ***12*** | ***-67*** |  | ***11*** | ***9*** | ***6*** | ***3*** | ***-69*** |
| ***All ages*** | ***69*** | ***49*** | ***37*** | ***32*** | ***-54*** |  | ***48*** | ***31*** | ***24*** | ***20*** | ***-59*** |
| ***Alcohol-specific (from 2000 onwards, ICD-10 E24.4, F10, G31.2, G62.1, G72.1, I42.6, K29.2, K70, K85.2, K86.0, Q86.0, R78.0, X45, X65, Y15)*** | | | | | | | | | | | |
| 0-14 |  |  | 0 | 0 |  |  |  |  | 0 | 0 |  |
| 15-29 |  |  | 2 | 2 |  |  |  |  | 1 | 1 |  |
| 30-44 |  |  | 26 | 21 |  |  |  |  | 11 | 11 |  |
| 45-59 |  |  | 83 | 58 |  |  |  |  | 35 | 27 |  |
| 60-74 |  |  | 84 | 69 |  |  |  |  | 28 | 24 |  |
| 75+ |  |  | 25 | 25 |  |  |  |  | 6 | 6 |  |
| ***0-64*** |  |  | ***36*** | ***26*** |  |  |  |  | ***15*** | ***12*** |  |
| ***All ages*** |  |  | ***39*** | ***30*** |  |  |  |  | ***15*** | ***13*** |  |

Mortality rates are rounded to the nearest whole number while % change shows the percentage change in actual (unrounded) rates.

Note that % change is not calculated, for a particular age group, when cause-specific mortality rates are consistently <5 per 100,000 population over time.

Table A2: Age-standardised cause-specific mortality rates (per 100,000 population) for some additional causes of death for males and females aged 0-64, 1980-82, 1991-92, 2000-02 and 2010-12 with % change showing overall percentage change in rates between 1981 and 2011. Rates are shown by Carstairs deprivation score (most to least deprived fifth)

|  | Males | | | | |  | Females | | | | |
| --- | --- | --- | --- | --- | --- | --- | --- | --- | --- | --- | --- |
|  | Years | | | | ***% Change*** |  | Years | | | | ***% Change*** |
|  | 1980-82 | 1991-92 | 2000-02 | 2010-12 | ***81-11*** |  | 1980-82 | 1991-92 | 2000-02 | 2010-12 | ***81-11*** |
| ***Lung cancer (ICD-9 162; ICD-10 C33-34)*** | | | | | | | | | | | |
| Most deprived | 87 | 71 | 57 | 37 | *-58* |  | 36 | 35 | 30 | 32 | *-11* |
| 2 | 64 | 54 | 38 | 26 | *-59* |  | 25 | 25 | 22 | 24 | *-4* |
| 3 | 62 | 42 | 31 | 21 | *-67* |  | 20 | 21 | 19 | 18 | *-8* |
| 4 | 44 | 34 | 24 | 17 | *-60* |  | 19 | 17 | 17 | 16 | *-16* |
| Least deprived | 40 | 24 | 15 | 11 | *-73* |  | 16 | 13 | 14 | 11 | *-34* |
| ***All Scotland*** | ***60*** | ***45*** | ***32*** | ***22*** | ***-64*** |  | ***23*** | ***22*** | ***20*** | ***20*** | ***-16*** |
| ***Breast cancer (ICD-9 175; ICD-10 C50)*** | | | | | | | | | | | |
| Most deprived |  |  |  |  |  |  | 31 | 24 | 21 | 19 | *-37* |
| 2 |  |  |  |  |  |  | 31 | 28 | 23 | 16 | *-50* |
| 3 |  |  |  |  |  |  | 33 | 30 | 21 | 18 | *-46* |
| 4 |  |  |  |  |  |  | 33 | 30 | 23 | 16 | *-53* |
| Least deprived |  |  |  |  |  |  | 30 | 29 | 20 | 17 | *-43* |
| ***All Scotland*** |  |  |  |  |  |  | ***32*** | ***29*** | ***22*** | ***17*** | ***-46*** |
| ***Prostate cancer (ICD-9 175; ICD-10 C50)*** | | | | | | | | | | | |
| Most deprived | 3 | 4 | 4 | 3 | *-* |  |  |  |  |  |  |
| 2 | 3 | 3 | 4 | 4 | *-* |  |  |  |  |  |  |
| 3 | 3 | 3 | 3 | 3 | *-* |  |  |  |  |  |  |
| 4 | 3 | 3 | 4 | 4 | *-* |  |  |  |  |  |  |
| Least deprived | 3 | 5 | 4 | 3 | *-* |  |  |  |  |  |  |
| ***All Scotland*** | ***3*** | ***3*** | ***4*** | ***3*** | ***-*** |  |  |  |  |  |  |
| ***Colorectal cancer (ICD-9 153, 154.0-154.1; ICD-10 C18-20)*** | | | | | | | | | | | |
| Most deprived | 14 | 16 | 14 | 9 | *-39* |  | 9 | 9 | 7 | 7 | *-25* |
| 2 | 14 | 14 | 10 | 9 | *-35* |  | 11 | 11 | 6 | 7 | *-38* |
| 3 | 12 | 14 | 11 | 9 | *-25* |  | 12 | 9 | 6 | 7 | *-42* |
| 4 | 12 | 13 | 12 | 6 | *-48* |  | 9 | 10 | 6 | 6 | *-37* |
| Least deprived | 13 | 14 | 11 | 6 | *-50* |  | 11 | 10 | 7 | 6 | *-46* |
| ***All Scotland*** | ***13*** | ***14*** | ***12*** | ***8*** | ***-40*** |  | ***11*** | ***10*** | ***6*** | ***7*** | ***-38*** |
| ***Stomach cancer (ICD-9 151; ICD-10 C16)*** | | | | | | | | | | | |
| Most deprived | 16 | 9 | 7 | 3 | *-82* |  | 5 | 5 | 3 | 2 | *-60* |
| 2 | 14 | 8 | 6 | 3 | *-78* |  | 5 | 4 | 3 | 2 | *-66* |
| 3 | 10 | 7 | 5 | 2 | *-78* |  | 5 | 2 | 1 | 1 | *-79* |
| 4 | 9 | 7 | 4 | 3 | *-67* |  | 4 | 3 | 1 | 1 | *-70* |
| Least deprived | 9 | 4 | 3 | 2 | *-80* |  | 3 | 2 | 2 | 1 | *-71* |
| ***All Scotland*** | 11 | 7 | 5 | 3 | ***-76*** |  | ***4*** | ***3*** | ***2*** | ***1*** | ***-69*** |
| ***Other cancers (all cancers excluding those above)*** | | | | | | | | | | | |
| Most deprived | 61 | 73 | 75 | 62 | *2* |  | 53 | 58 | 52 | 42 | *-22* |
| 2 | 55 | 61 | 63 | 53 | *-5* |  | 52 | 52 | 44 | 36 | *-30* |
| 3 | 51 | 55 | 56 | 46 | *-9* |  | 51 | 46 | 45 | 36 | *-29* |
| 4 | 53 | 57 | 55 | 44 | *-17* |  | 49 | 43 | 39 | 33 | *-33* |
| Least deprived | 45 | 47 | 46 | 38 | *-16* |  | 40 | 44 | 35 | 33 | *-19* |
| ***All Scotland*** | ***53*** | ***58*** | ***59*** | ***48*** | ***-10*** |  | ***49*** | ***48*** | ***43*** | ***36*** | ***-28*** |
| ***Suicide (excluding alcohol and drug poisonings, ICD-9 E950.6-959, E980.6-989; ICD-10 X64-84, Y87.0, Y16-34, Y87.2)*** | | | | | | | | | | | |
| Most deprived | 20 | 21 | 29 | 24 | *20* |  | 4 | 4 | 5 | 6 | *27* |
| 2 | 15 | 15 | 25 | 19 | *32* |  | 4 | 4 | 4 | 4 | *-2* |
| 3 | 14 | 13 | 21 | 17 | *23* |  | 5 | 5 | 4 | 4 | *-30* |
| 4 | 14 | 16 | 18 | 17 | *18* |  | 4 | 2 | 3 | 5 | *10* |
| Least deprived | 11 | 15 | 13 | 12 | *8* |  | 5 | 3 | 3 | 2 | *-50* |
| ***All Scotland*** | ***15*** | ***16*** | ***21*** | ***18*** | ***22*** |  | ***5*** | ***3*** | ***4*** | ***4*** | ***-10*** |
| ***Accidents (excluding alcohol and drug poisonings, ICD-9 E800-849, E861-929; ICD-10 V01-X39, X46-59, Y85, Y86)*** | | | | | | | | | | | |
| Most deprived | 47 | 30 | 23 | 16 | *-66* |  | 13 | 13 | 7 | 4 | *-69* |
| 2 | 39 | 27 | 21 | 11 | *-72* |  | 12 | 8 | 5 | 3 | *-74* |
| 3 | 37 | 24 | 19 | 12 | *-66* |  | 12 | 9 | 6 | 4 | *-61* |
| 4 | 35 | 24 | 19 | 11 | *-67* |  | 11 | 6 | 6 | 3 | *-72* |
| Least deprived | 29 | 18 | 13 | 11 | *-64* |  | 9 | 7 | 4 | 3 | *-69* |
| ***All Scotland*** | ***37*** | ***25*** | ***19*** | ***12*** | ***-67*** |  | ***11*** | ***9*** | ***6*** | ***3*** | ***-69*** |
| ***Alcohol-specific (from 2000 onwards, ICD-10 E24.4, F10, G31.2, G62.1, G72.1, I42.6, K29.2, K70, K85.2, K86.0, Q86.0, R78.0, X45, X65, Y15)*** | | | | | | | | | | | |
| Most deprived |  |  | 80 | 52 |  |  |  |  | 26 | 22 |  |
| 2 |  |  | 45 | 33 |  |  |  |  | 19 | 15 |  |
| 3 |  |  | 28 | 24 |  |  |  |  | 13 | 12 |  |
| 4 |  |  | 22 | 17 |  |  |  |  | 10 | 8 |  |
| Least deprived |  |  | 11 | 10 |  |  |  |  | 7 | 6 |  |
| ***All Scotland*** |  |  | ***36*** | ***26*** |  |  |  |  | ***15*** | ***12*** |  |

Mortality rates are rounded to the nearest whole number while % change shows the percentage change in actual (unrounded) rates

Table A3: Average number of deaths per year over each period

|  | 1980-82 | 1991-92 | 2000-02 | 2010-12 |
| --- | --- | --- | --- | --- |
| *Census population* | *5,178,248* | *5,106,135* | *5,062,011* | *5,295,403* |
| Cause |  |  |  |  |
| All causes | 63,417 | 60,249 | 57,197 | 53,936 |
| Ischaemic heart disease | 18,078 | 16,469 | 11,854 | 7,693 |
| Cancer | 13,834 | 14,908 | 14,977 | 15,515 |
| Stroke | 9,049 | 7,828 | 6,678 | 4,597 |
| Chronic lower respiratory diseases | 2,472 | 2,547 | 2,983 | 3,036 |
| Influenza and pneumonia | 4,009 | 3,738 | 2,407 | 2,141 |
| Dementia and Alzheimer disease | 323 | 699 | 1,743 | 3,067 |
| Alcohol-related | 577 | 595 | 1,382 | 1,210 |
| Drug-related | 287 | 292 | 528 | 710 |
| Suicide | 725 | 731 | 861 | 823 |
| Accidents | 2,154 | 1,571 | 1,282 | 1,492 |
| Lung cancer | 4,041 | 4,240 | 3,947 | 4,136 |
| Breast cancer | 1,199 | 1,260 | 1,114 | 1,037 |
| Prostate cancer | 468 | 669 | 770 | 876 |
| Colorectal cancer | 1,693 | 1,710 | 1,583 | 1,546 |
| Stomach cancer | 1,075 | 826 | 646 | 487 |
| Other cancers | 5,357 | 6,205 | 6,918 | 7,433 |
| Suicide (excluding alcohol and drug poisonings) | 467 | 482 | 604 | 533 |
| Accidents (excluding alcohol and drug poisonings) | 2,117 | 1,550 | 1,264 | 1,152 |
| Alcohol-specific (from 2000 onwards) | - | - | 1,228 | 1,092 |
